# Lorlatinib Versus Pemetrexed-Based Chemotherapy in Patients With ALK-rearranged NSCLC Previously Treated With Alectinib

**DOI:** 10.1016/j.jtocrr.2022.100311

**Published:** 2022-03-17

**Authors:** Yuki Takeyasu, Tatsuya Yoshida, Ken Masuda, Yuji Matsumoto, Yuki Shinno, Yusuke Okuma, Yasushi Goto, Hidehito Horinouchi, Noboru Yamamoto, Yuichiro Ohe

**Affiliations:** a Department of Thoracic Oncology, National Cancer Center Hospital, Tokyo, Japan; b Course of Advanced Clinical Research of Cancer, Juntendo University Graduate School of Medicine, Tokyo, Japan

**Keywords:** Non–small cell lung cancer, Anaplastic lymphoma kinase, Lorlatinib, Chemotherapy, Pemetrexed

## Abstract

**Introduction:**

Lorlatinib (LOR) or pemetrexed-based chemotherapy (PEM) is the standard treatment after failure of a second-generation ALK tyrosine kinase inhibitor, such as alectinib, in patients with ALK-positive NSCLC. Nevertheless, there have been few data on the clinical outcomes of these treatments after alectinib failure.

**Methods:**

We retrospectively analyzed patients with ALK-rearranged NSCLC who received LOR (LOR group) or PEM (PEM group) as post-treatment after alectinib failure between December 2012 and August 2020.

**Results:**

Among 90 patients who experienced disease progression during alectinib treatment, 38 of them received either PEM (*n* = 22) or LOR (*n* = 16) as subsequent treatment. The objective response rate and the median progression-free survival were similar in the PEM and LOR groups (objective response rate: 45% versus 44%, *p* = 0.92; median progression-free survival: 6.9 mo versus 6.2 mo, *p* = 0.83, respectively). Disease progression during treatment occurred in 22 patients with PEM and 14 patients with LOR. The central nervous system (CNS) was the most common site of progression in both groups. In patients without CNS metastasis at baseline, the cumulative incidence rate of CNS progression was lower over time in the LOR group compared with the PEM group (*p* = 0.045), whereas in patients with CNS metastasis at baseline, there were no significant differences in cumulative incidence rate of CNS progression between both groups (*p* = 0.43).

**Conclusions:**

Clinical outcomes of PEM and LOR after failure of alectinib were similar in patients with ALK-positive NSCLC.

## Introduction

ALK rearrangements are found in approximately 2% to 5% of NSCLC. ALK tyrosine kinase inhibitors (TKIs) were found to have statistical significant progression-free survival (PFS) prolongation compared with platinum chemotherapy in several phase 3 studies.^1^, ^2^, ^3^ Second-generation ALK TKIs, alectinib and brigatinib, were found to have significantly better activity and safety profile compared with crizotinib, a first-generation ALK TKI, in treatment naive advanced NSCLC patients with ALK rearrangement.^4^, ^5^, ^6^ Despite such improvements in clinical outcomes in ALK-rearranged NSCLC, almost all patients acquired resistance to ALK TKIs.

Lorlatinib (LOR), a third-generation ALK TKI, was specifically developed to have broad activity against ALK-resistant mutations. In the open-label, phase 1/2 study (NCT01970865), LOR was found to have intracranial and extracranial responses in patients with advanced NSCLC harboring ALK rearrangement, who experienced tumor progression during prior ALK TKI therapy, including alectinib.^7^^,^^8^ Pemetrexed-based cytotoxic chemotherapy has been reported to be superior to other anticancer agents and has been the other key treatment in ALK-positive NSCLC.^9^ Therefore, LOR or pemetrexed-based chemotherapy (PEM) is the standard treatment after failure of second-generation ALK TKIs, such as alectinib. Nevertheless, it remains unclear which treatment should be selected in patients with ALK-positive NSCLC who experienced failure of second-generation ALK TKI, especially alectinib.

In this study, we aimed to evaluate the efficacy of LOR or PEM as a post-alectinib treatment and the difference of progression patterns during treatment with LOR or PEM.

## Materials and Methods

### Patients

Patients with ALK-rearranged NSCLC who received LOR (LOR group [100 mg orally once daily]) or PEM (PEM group [500 mg/m^2^]: pemetrexed alone or combination with a platinum agent) as post-alectinib treatment between December 2012 and May 2020 at the National Cancer Center Hospital were included. The cutoff date for our analysis was March 30, 2021. Medical records, including patient characteristics and clinical outcomes, were retrospectively reviewed. The *ALK* gene rearrangement was identified by immunohistochemistry (ALK Detection Kit, Nichirei Bioscience, Tokyo, Japan; DF53, Roche, Basel, Switzerland; and 5A4, Abcam, Cambridge, United Kingdom), fluorescence in situ hybridization (Vysis ALK Break Apart FISH Probe Kit, Abbott Molecular, Abbott Park, IL), reverse-transcriptase polymerase chain reaction analysis, and next-generation sequencing (Oncomine Dx Target Test, Thermo Fisher Scientific, Waltham, MA).

### Statistical Analysis

The tumor response was assessed according to the Response Evaluation Criteria for Solid Tumors, version 1.1.^10^ The objective response rate (ORR) and disease control rate were defined as the proportion of patients who had an objective best response (complete or partial response) or disease control (complete response, partial response, or stable disease). Treatment-related adverse events (AEs) were graded according to the Common Terminology Criteria for Adverse Events, version 4.03. Differences in baseline characteristics and response rate between groups were compared using the chi-square or Fisher’s exact test for categorical data, as appropriate. PFS, overall survival (OS), and follow-up period were estimated with the Kaplan-Meier method, and comparisons were analyzed using the log-rank test. PFS was calculated from the date of initiation of LOR or PEM treatment to disease progression, death, or the last follow-up visit. OS was calculated from the start of LOR or PEM to death or the last follow-up visit. Early progression during alectinib was defined as disease progression within 12 months from the start of alectinib treatment because the PFS of crizotinib was 10 to 12 months, based on the results of clinical trials.^1^ The cumulative incidence rate of central nervous system (CNS)/systemic progression was analyzed using the competing risk method. Each event was defined as CNS/systemic progression, or other progression, or death, and patients were censored when the earliest of the events occurred. All *p* values less than 0.05 were considered to indicate statistical significance. All data were analyzed using JMP Pro version 13.1.0 (SAS Institute, Cary, NC). This study was approved by the institutional ethics committee of the National Cancer Center Hospital.

## Results

### Patient Characteristics

Between December 2012 and August 2020, a total of 164 patients with advanced NSCLC harboring ALK rearrangement received alectinib. Among these patients, 90 experienced disease progression during alectinib treatment and 38 received either PEM (PEM group, *n* = 22) or LOR (LOR group, *n* = 16) as subsequent treatment. Baseline patient characteristics are found in Table 1. Histology in all patients was adenocarcinoma, and more than half of the patients in both groups were never smokers. Eight patients (36%) in the PEM group and four (25%) in the LOR group received crizotinib before alectinib. Meanwhile, the prevalence of CNS metastasis before treatment in the PEM group was less than that in the LOR group (27% versus 55%).

**Table 1:** Baseline Patient Characteristics

Patients’ Characteristics	Pem-Based CTx *n* = 22	Lorlatinib *n* = 16	*p* Value
Age at diagnosis of advanced disease			
Median (range), y	49 (19–77)	57 (32–75)	
Sex, *n* (%)			0.4006
Male	8 (36)	8 (50)	
Female	14 (64)	8 (50)	
Smoking history, *n* (%)			0.2147
Never	17 (77)	9 (56)	
Light (≤10 pack years)	3 (14)	2 (13)	
Heavy (>10 pack years)	2 (9)	5 (31)	
Histopathology, *n* (%)			1.00
Adenocarcinoma	22 (100)	16 (100)	
Stage at diagnosis, *n* (%)			0.3845
III–IV	20 (91)	13 (81)	
Recurrence	2 (9)	3 (19)	
ECOG performance states, *n* (%)			0.7353^a^
0	13 (59)	5 (31)	
1	9 (41)	9 (56)	
2–	0	2 (13)	
Brain metastasis before treatment, *n* (%)			0.1560
Absent	12 (55)	5 (31)	
Present	10 (45)	11 (69)	
Treatment line of therapy, *n* (%)			0.0970
2	12 (55)	11 (69)	
3	9 (41)	2 (13)	
4–	1 (5)	3 (19)	
Number of prior ALK TKIs, *n* (%)			0.2372
1	14 (64)	12 (75)	
2	8 (36)	4 (25)	
Median PFS, mo (95% CI)			
Alectinib	10.9 (6.8–16.0)	14.0 (8.4–14.7)	0.0921

CI, confidence interval; CTx, chemotherapy; ECOG, Eastern Cooperative Oncology Group; Pem, pemetrexed; PFS, progression-free survival; PS, performance status; TKI, tyrosine kinase inhibitor.

a The statistical test was between PS 0 to 1 and PS 2.

In the PEM group, the cytotoxic regimens included the following: platinum/pemetrexed (13 of 22 patients, 59%), platinum/pemetrexed/bevacizumab (6 of 22 patients, 27%), pemetrexed monotherapy (2 of 22 patients, 9%), and platinum/pemetrexed/pembrolizumab (1 of 22 patients, 5%).

### Efficacy of LOR and PEM

**Figure 1:**
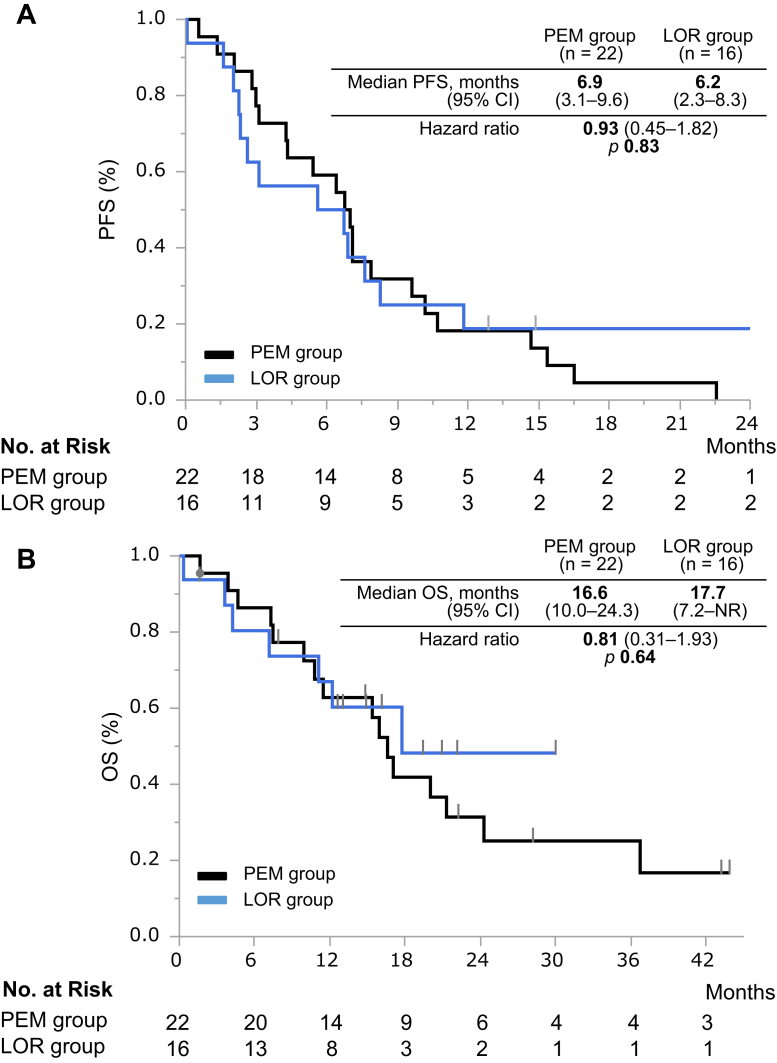
(*A*) PFS and (*B*) OS of patients with ALK-rearranged non-small cell lung cancer previously treated with alectinib. CI, confidence interval; LOR, lorlatinib; No., number; NR, not reported; OS, overall survival; PEM, pemetrexed-based chemotherapy; PFS, progression-free survival.

**Figure 2:**
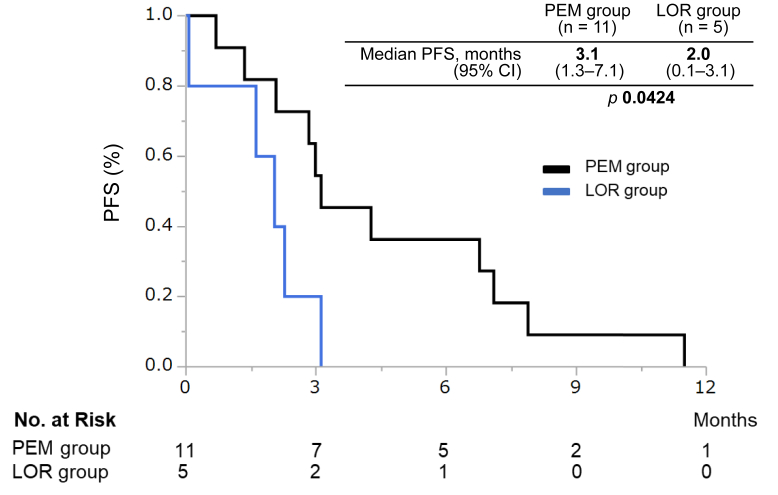
PFS of patients who experienced early progression (within 1 y) during alectinib as the initial ALK TKI. CI, confidence interval; LOR, lorlatinib; No., number; PEM, pemetrexed-based chemotherapy; PFS, progression-free survival; TKI, tyrosine kinase inhibitor.

The median follow-up time was 15.7 months (95% confidence interval [CI]: 7.9–21.3) in the PEM group and 12.8 months (95% CI: 4.2–17.7) in the LOR group. In the PEM group (*n* = 22), an objective response was achieved in 10 patients (ORR = 45%, 95% CI: 27–65); in the LOR group (*n* = 16), objective response was achieved in seven patients (ORR = 44%, 95% CI: 23–67). The ORR was similar in both groups (*p* = 0.9169). The disease control rate was achieved in 82% (95% CI: 61–93) of the PEM group and 63% (95% CI: 39–82) of the LOR group with no significant difference (*p* = 0.1818) (Table 2). The median PFS (mPFS) was also similar in both groups (PEM versus LOR: 6.9 mos [95% CI: 3.1–9.6] versus 6.2 mo [95% CI: 2.3–8.3], hazard ratio [HR] = 0.93, 95% CI: 0.45–1.82, *p* = 0.83) (Fig. 1*A*). In the population who experienced early progression (within 1 y) while on alectinib as the initial ALK TKI, the mPFS in the LOR group was significantly longer than the PEM group (PEM versus LOR: 3.1 mo [95% CI: 1.3–7.1] versus 2.0 mo [95% CI: 0.1–3.1], respectively, *p* = 0.042; Fig. 2). There were no significant differences in OS after alectinib failure between both groups (PEM versus LOR: 16.6 mo [95% CI: 10.0–24.3] versus 17.7 mo [95% CI: 7.2–not reached], HR = 1.05, 95% CI: 0.39–2.64, *p* = 0.9245) (Fig. 1*B*).

**Table 2:** Tumor Responses

Variables	Pem-Based CTx	Lorlatinib	*p* Value
All patients, *n*	22	16	
Best overall response, *n* (%)			
CR	0	0	
PR	10 (45)	7 (44)	
SD	8 (36)	3 (19)	
PD	4 (18)	4 (25)	
NE	0	2 (13)	
Clinical benefit, %			
ORR	45	44	0.9169
DCR	82	63	0.1818

CR, complete response; CTx, chemotherapy; DCR, disease control rate; NE; not evaluable; ORR, objective response rate; PD, progression disease; Pem, pemetrexed; PR, partial response; SD, stable disease.

### Safety of LOR and PEM

Treatment-related AEs are found in Supplementary Table 1. Overall, 95% and 81% of patients in the PEM and LOR group, respectively, had at least one treatment-related AE. AEs of grade 3 or higher occurred in 14% of the patients in the PEM group and 38% of those in the LOR group (*p* = 0.0876). In the LOR group, dose interruptions and reductions owing to AEs occurred in three (19%) and in four (25%) patients, respectively. In the PEM group, no dose interruption or dose reduction occurred.

### Progression Patterns During LOR and PEM

**Figure 3:**
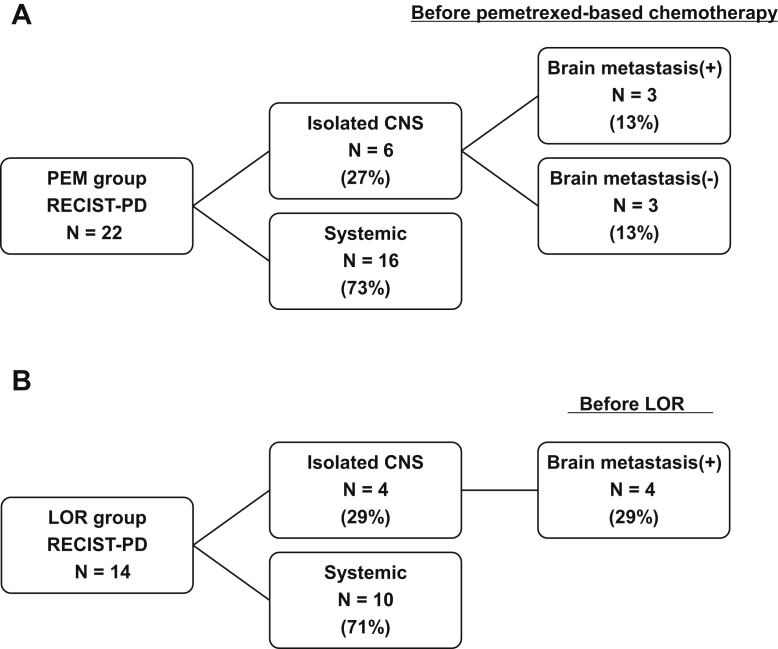
Progression patterns at the RECIST-PD status after (*A*) PEM or (*B*) LOR. CNS, center nervous system; LOR, lorlatinib; PEM, pemetrexed-based chemotherapy; RECIST-PD, Response Evaluation Criteria for Solid Tumors—progression disease.

**Figure 4:**
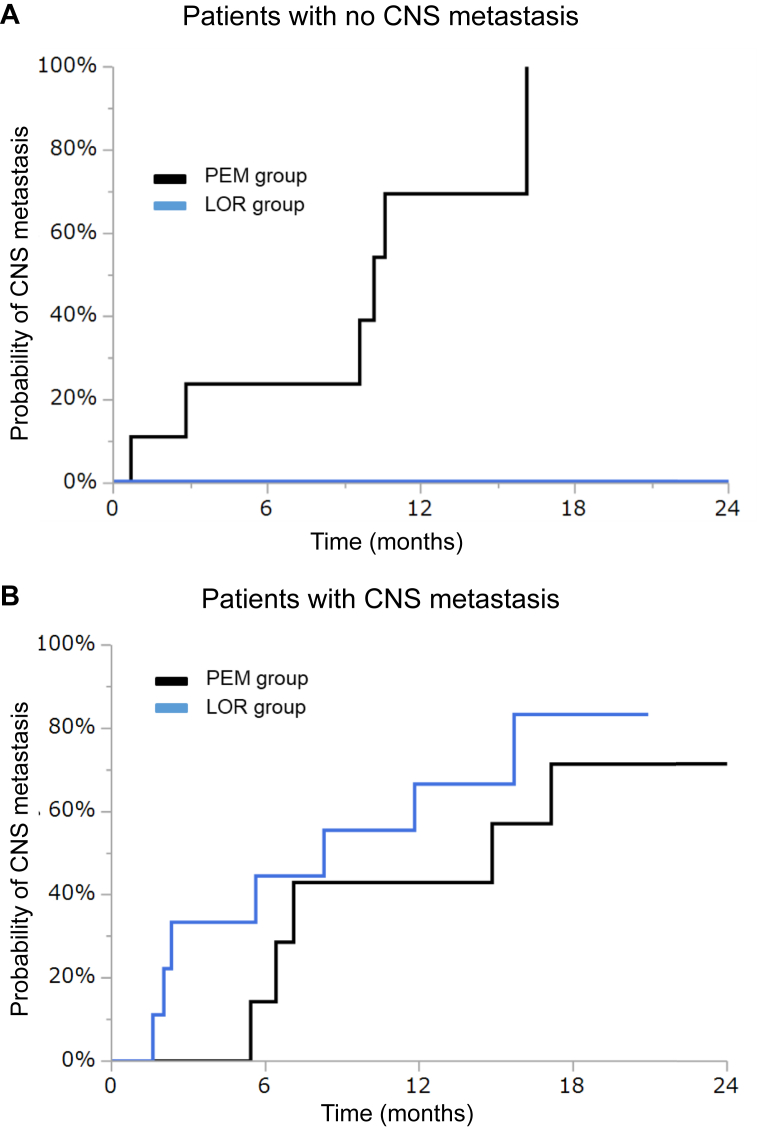
Cumulative incidence of CNS progression. (*A*) PEM group versus LOR group in patients with no CNS metastasis. (*B*) PEM group versus LOR group in patients with CNS metastasis. CNS, central nervous system; LOR, lorlatinib; PEM, pemetrexed-based chemotherapy.

Progression patterns in the PEM and LOR groups are summarized in Figure 3*A* and *B*. A total of 22 patients in the PEM group and 14 in the LOR group had disease progression during treatment. A total of 17 patients (77%) in the PEM group and five patients (31%) in the LOR group received subsequent treatment. Of these patients, six (35%) in the PEM group received LOR and four (80%) in the LOR group received PEM (Supplementary Figs. 1 and 2). The CNS was the most common site of progression in eight patients (36%) in the PEM and in seven patients (50%) in the LOR groups. CNS progression in both groups was identified more frequently in patients with CNS metastasis at baseline compared with those without (PEM group: 40% versus 33%, *p* = 0.7463; LOR group: 66.7% versus 20%, *p* = 0.086). In patients without CNS metastasis at baseline, the cumulative incidence rate of CNS progression was lower over time in the LOR group compared with the PEM group (log-rank test, *p* = 0.045; Fig. 4*A*). Meanwhile, in patients with CNS metastasis at baseline, there were no significant differences in the cumulative incidence rate of CNS progression between both groups (log-rank test, *p* = 0.43; Fig. 4*B*). The prevalence of systemic progression during treatment was similar between the two groups (PEM versus LOR: 73% [16 of 22] versus 71% [10 of 14], *p* = 0.93).

## Discussion

We analyzed clinical outcomes of post-treatment after alectinib failure in ALK-rearranged NSCLC and revealed similar ORR and PFS between the PEM and LOR groups ([LOR group] mPFS = 6.2 mo, ORR = 44%; and [PEM group] mPFS = 6.9, ORR = 45%), which were consistent with previous reports.^9^^,^^11^

Data on the efficacy of other ALK TKIs after the failure of second-generation ALK TKIs, such as alectinib. in ALK-rearranged NSCLC are limited. In phase 2 studies of patients with ALK-rearranged NSCLC who were previously treated with alectinib, ceritinib resulted in an ORR of 25% and mPFS of 3.7 months^12^ and brigatinib resulted in an ORR of 34% and mPFS of 7.3 months.^13^ Lorlatinib was found to have not only extracranial but also intracranial activity in other ALK TKIs, including second-generation ALK TKIs, in refractory patients with ALK-rearranged NSCLC with or without CNS metastases (mPFS = 6.6 mo, ORR = 39.6%, intracranial ORR = 56.1%).^11^

Pemetrexed-based chemotherapy is effective in patients with ALK-rearranged NSCLC who were treatment naive and previously treated, and it has been the optimal treatment for patients who experienced disease progression while on second-generation ALK TKIs.^1^^,^^2^ Updated results from the ALEX study revealed that 38.1% of the patients who experienced progression during alectinib received ALK TKIs and 26.2% received PEM. The retrospective study revealed that ORR of platinum/pemetrexed was 29.7% and the mPFS was 4.3 months, which were comparable with the results of our study.^9^

Our study revealed that among patients who experienced early progression (within 1 y) during alectinib treatment, PFS in the LOR group was significantly shorter than that in the PEM group. The preclinical studies revealed that LOR had more potent activity against nonmutant ALK than first- and second-generation ALK TKIs.^14^ Moreover, the efficacy of LOR was associated with the presence of ALK-resistant mutations, which were related to continual ALK dependence.^15^ In addition, in an exploratory analysis of a phase 2 trial of LOR, LOR was found to have greater efficacy in patients with *ALK*-resistant mutations compared with patients without *ALK*-resistant mutations.^16^ Therefore, these results could suggest that tumors with early progression during alectinib treatment were involved in ALK-independent resistance. In patients with such tumors, PEM was a better treatment than LOR.

There were several limitations in our study. First, our study was a small, single-center retrospective study. The frequency of imaging was at the physician’s discretion and was not designed to directly compare the efficacy of LOR to PEM. Nevertheless, all patients underwent regular outpatient follow-up every 1 to 2 months and computed tomography or magnetic resonance imaging every 3 to 6 months. In addition, the number of patients with intracranial lesions was higher in the LOR group compared with the PEM group, because data on the efficacy of LOR on intracranial lesions have influence on regimen selection.^11^ Indeed, LOR was active against CNS metastasis and reduced the progression of CNS lesions. Nevertheless, in our study, the PFS of PEM or LOR did not differ in both patients with and without CNS metastasis at baseline (with: HR = 0.91, 95% CI: 0.25–2.65)/(without: HR = 0.62, 95% CI: 0.22–1.67) (Supplementary Fig. 3*A* and *B*). Second, sequential ALK TKI treatments, lines of therapy, and regimens of PEM were heterogeneous, which could potentially influence data on the efficacy of LOR and PEM. Nevertheless, even when the PEM group was adjusted to a homogeneous population excluding patients treated with pemetrexed monotherapy and platinum-pemetrexed plus bevacizumab or pembrolizumab, clinical outcomes were similar (PEM group versus LOR group: PFS = 6.4 [95% CI: 2.1–7.9] versus 6.2 [95% CI: 2.3–8.3], *p* = 0.56, OS = 16.6 [7.3–36.7] versus 17.7 [7.2–not reported], *p* = 0.96) (Supplementary Fig. 4). This result reconfirmed the result from our study that pemetrexed is a core drug for ALK-rearranged NSCLC. Finally, ALK-resistant mutation that affects the activity of LOR was not assessable owing to the accessibility of tissues after alectinib failure. LOR was found to have greater efficacy in patients with *ALK*-resistant mutations, such as G1202R and I1171N, which cause resistance to alectinib compared with patients without *ALK* mutations.

In conclusion, our study suggested that the efficacy of PEM and LOR is comparable in advanced ALK-rearranged NSCLC after second-generation ALK TKI failure. Nevertheless, the efficacy of LOR might be limited in patients who experience early progression (within 1 y) during alectinib treatment. Further investigation on the treatment sequence, including ALK TKIs and PEM based on the presence of resistance mechanism of ALK TKIs, is needed in prospective clinical trials.

## CRediT Authorship Contribution Statement

**Yuki Takeyasu, Tatsuya Yoshida:** Study concepts, Study design, Quality control of data and algorithms, Data analysis and interpretation, Statistical analysis, Manuscript preparation, Manuscript editing.

**Yuki Takeyasu:** Data acquisition.

**Yuki Takeyasu, Tatsuya Yoshida, Ken Masuda, Yuki Shinno, Yusuke Okuma, Noboru Yamamoto, Yasushi Yatabe, Yuichiro Ohe:** Manuscript review.

## References

[1] Solomon B.J., Mok T., Kim D.W. (2014). First-line crizotinib versus chemotherapy in ALK-positive lung cancer. N Engl J Med.

[2] Soria J.C., Tan D.S.W., Chiari R. (2017). First-line ceritinib versus platinum-based chemotherapy in advanced ALK-rearranged non-small-cell lung cancer (ASCEND-4): a randomised, open-label, phase 3 study. Lancet.

[3] Wu Y.L., Lu S., Lu Y. (2018). Results of PROFILE 1029, a phase III comparison of first-line crizotinib versus chemotherapy in East Asian patients with ALK-positive advanced non-small cell lung cancer. J Thorac Oncol.

[4] Hida T., Nokihara H., Kondo M. (2017). Alectinib versus crizotinib in patients with ALK-positive non-small-cell lung cancer (J-ALEX): an open-label, randomised phase 3 trial. Lancet.

[5] Peters S., Camidge D.R., Shaw A.T. (2017). Alectinib versus crizotinib in untreated ALK-positive non-small-cell lung cancer. N Engl J Med.

[6] Camidge D.R., Kim H.R., Ahn M.J. (2018). Brigatinib versus crizotinib in ALK-positive non-small-cell lung cancer. N Engl J Med.

[7] Shaw A.T., Felip E., Bauer T.M. (2017). Lorlatinib in non-small-cell lung cancer with ALK or ROS1 rearrangement: an international, multicentre, open-label, single-arm first-in-man phase 1 trial. Lancet Oncol.

[8] Solomon B.J., Besse B., Bauer T.M. (2018). Lorlatinib in patients with ALK-positive non-small-cell lung cancer: results from a global phase 2 study. Lancet Oncol.

[9] Lin J.J., Schoenfeld A.J., Zhu V.W. (2020). Efficacy of platinum/pemetrexed combination chemotherapy in ALK-positive NSCLC refractory to second-generation ALK inhibitors. J Thorac Oncol.

[10] Eisenhauer E.A., Therasse P., Bogaerts J. (2009). New response evaluation criteria in solid tumours: revised RECIST guideline (version 1.1). Eur J Cancer.

[11] Felip E., Shaw A.T., Bearz A. (2021). Intracranial and extracranial efficacy of lorlatinib in patients with ALK-positive non-small-cell lung cancer previously treated with second-generation ALK TKIs. Ann Oncol.

[12] Hida T., Seto T., Horinouchi H. (2018). Phase II study of ceritinib in alectinib-pretreated patients with anaplastic lymphoma kinase-rearranged metastatic non-small-cell lung cancer in Japan: ASCEND-9. Cancer Sci.

[13] Nishio M., Yoshida T., Kumagai T. (2021). Brigatinib in Japanese patients with ALK-positive NSCLC previously treated with alectinib and other tyrosine kinase inhibitors: outcomes of the phase 2 J-ALTA trial. J Thorac Oncol.

[14] Zou H.Y., Friboulet L., Kodack D.P. (2015). PF-06463922, an ALK/ROS1 inhibitor, overcomes resistance to first and second generation ALK inhibitors in preclinical models. Cancer Cell.

[15] Gainor J.F., Dardaei L., Yoda S. (2016). Molecular mechanisms of resistance to first- and second-generation ALK inhibitors in ALK-rearranged lung cancer. Cancer Discov.

[16] Shaw A.T., Solomon B.J., Besse B. (2019). ALK resistance mutations and efficacy of lorlatinib in advanced anaplastic lymphoma kinase-positive non-small-cell lung cancer. J Clin Oncol.

