# Supplementary material for: Lorlatinib Versus Pemetrexed-Based Chemotherapy in Patients With ALK-rearranged NSCLC Previously Treated With Alectinib
Source: JTO Clin Res Rep. 2022 Mar 17;3(5):100311. doi: 10.1016/j.jtocrr.2022.100311 (PMC9046446; doi:10.1016/j.jtocrr.2022.100311)
Supplement: Supplementary — Figs [file mmc1.pptx]

## Slide 1
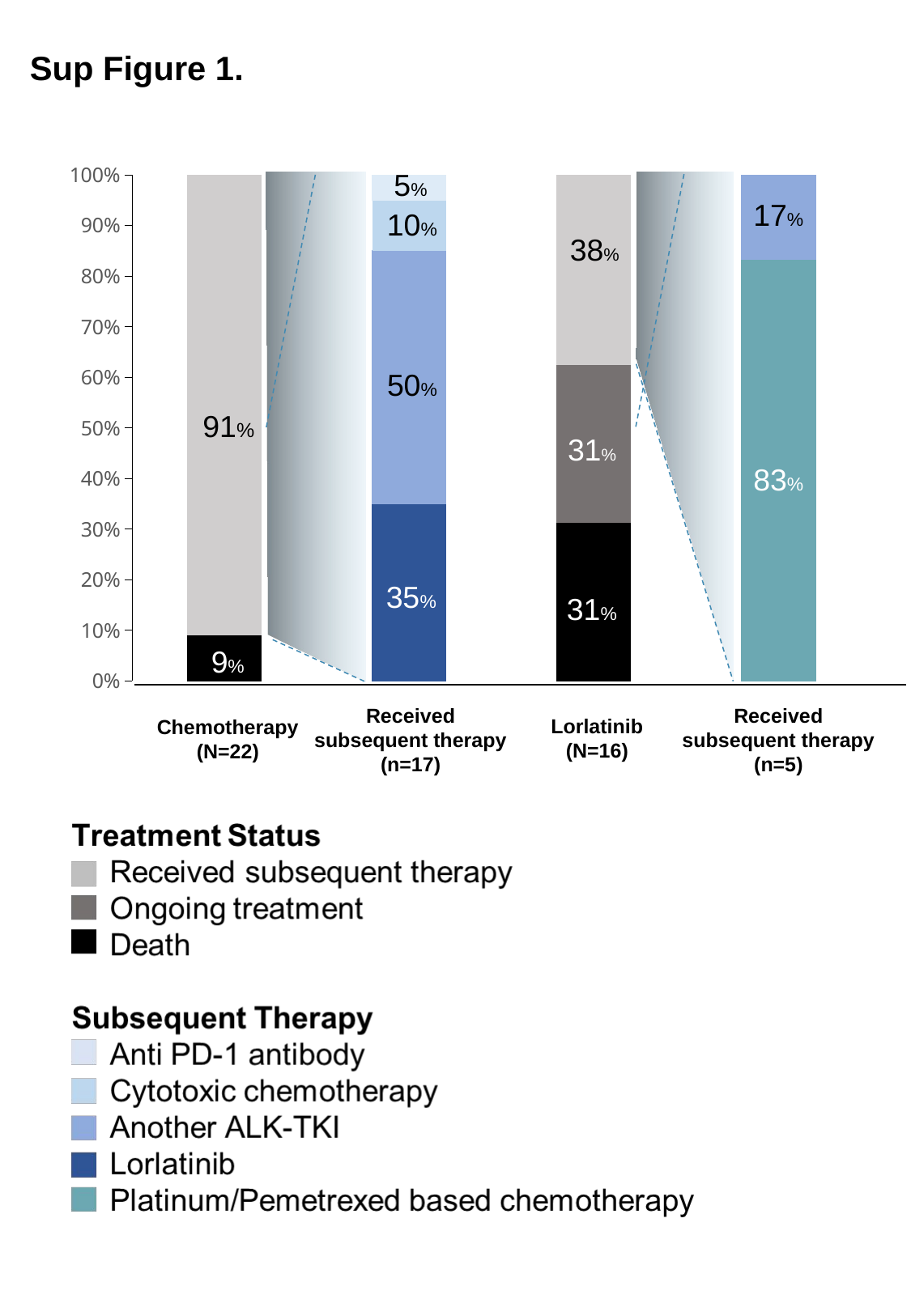

Sup Figure 1.
### Chart
| Category | 系列 1 | 系列 2 | 系列 3 | 系列 4 |
|---|---|---|---|---|
| Chemotherapy | 2.0 | 0.0 | 20.0 | None |
| Received subsequent therapy | 7.0 | 10.0 | 2.0 | 1.0 |
| Lorlatinib | 5.0 | 5.0 | 6.0 | None |
| Received subsequent therapy | 5.0 | 1.0 | None | None |5%
17%
10%
38%
50%
91%
31%
83%
35%
31%
9%
Received subsequent therapy
(n=17)
Received subsequent therapy
(n=5)
Lorlatinib
(N=16)
Chemotherapy
(N=22)

## Slide 2
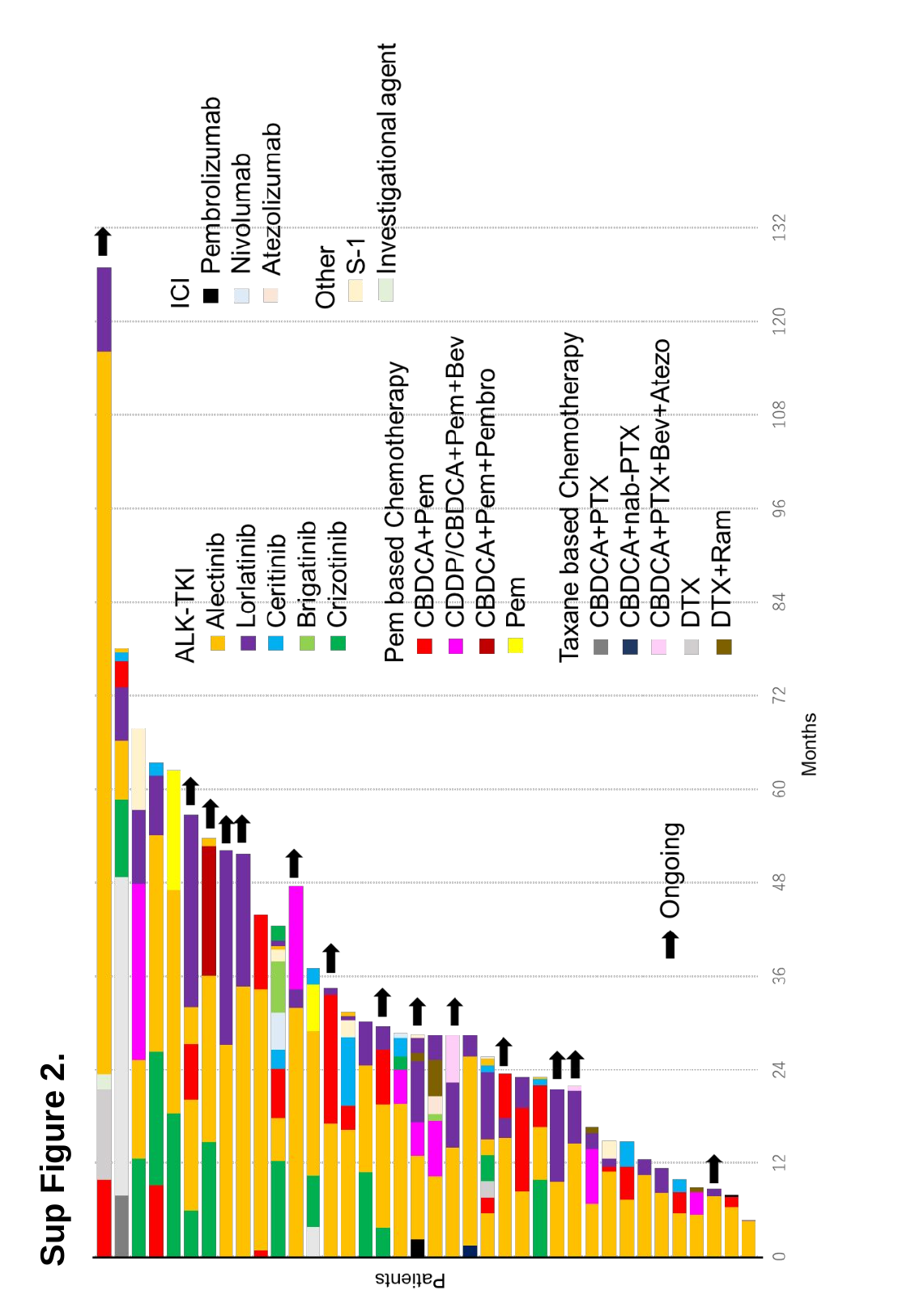

Sup Figure 2.

## Slide 3
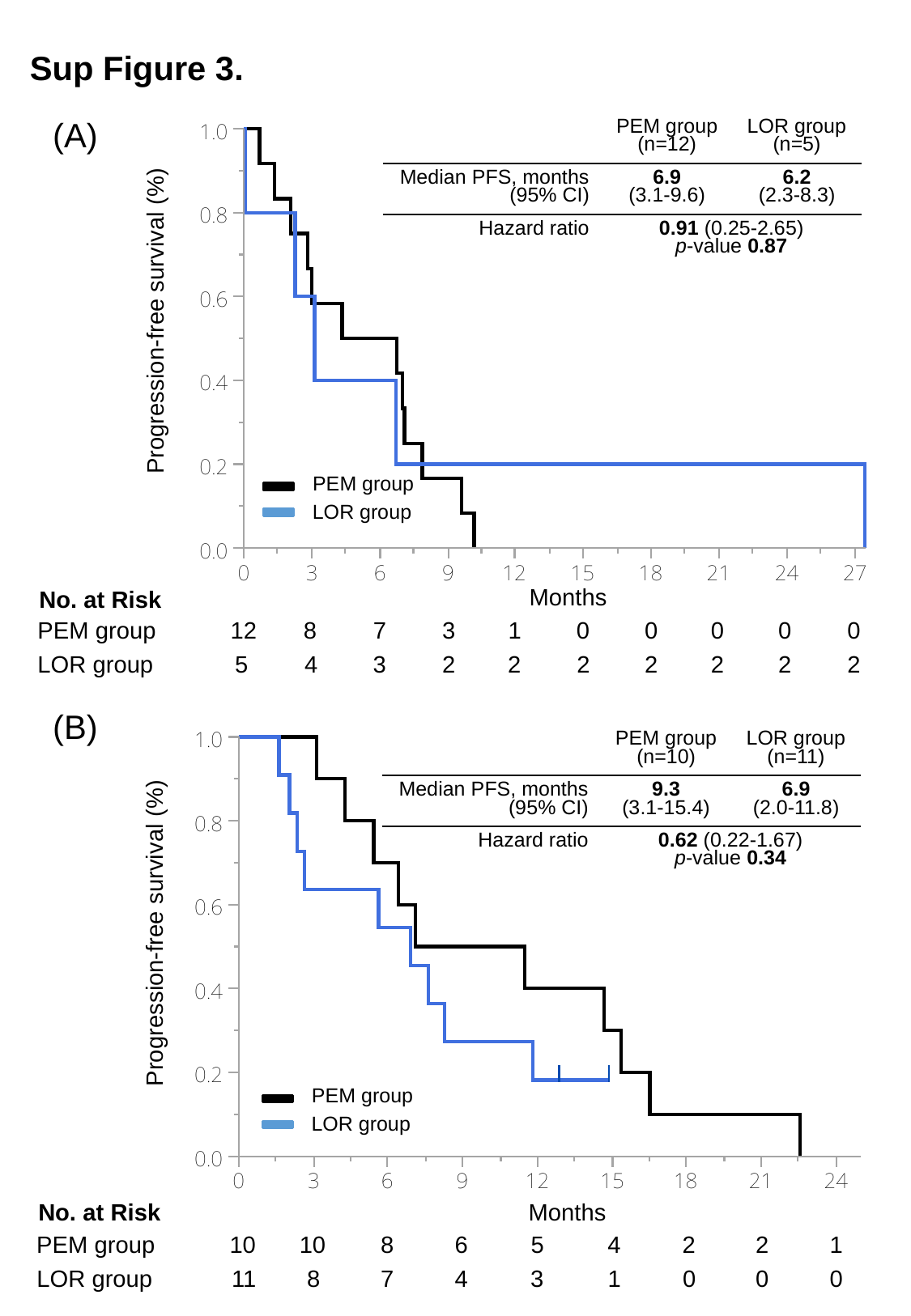

Sup Figure 3.
(A)
| | PEM group (n=12) | LOR group (n=5) |
| --- | --- | --- |
| Median PFS, months (95% CI) | 6.9 (3.1-9.6) | 6.2 (2.3-8.3) |
| Hazard ratio | 0.91 (0.25-2.65) p-value 0.87 | |
Progression-free survival (%)
PEM group
LOR group
Months
No. at Risk
PEM group
12
8
7
3
1
0
0
0
0
0
LOR group
5
4
3
2
2
2
2
2
2
2
(B)
| | PEM group (n=10) | LOR group (n=11) |
| --- | --- | --- |
| Median PFS, months (95% CI) | 9.3 (3.1-15.4) | 6.9 (2.0-11.8) |
| Hazard ratio | 0.62 (0.22-1.67) p-value 0.34 | |
Progression-free survival (%)
PEM group
LOR group
Months
No. at Risk
PEM group
10
10
8
6
5
4
2
2
1
LOR group
11
8
7
4
3
1
0
0
0

## Slide 4
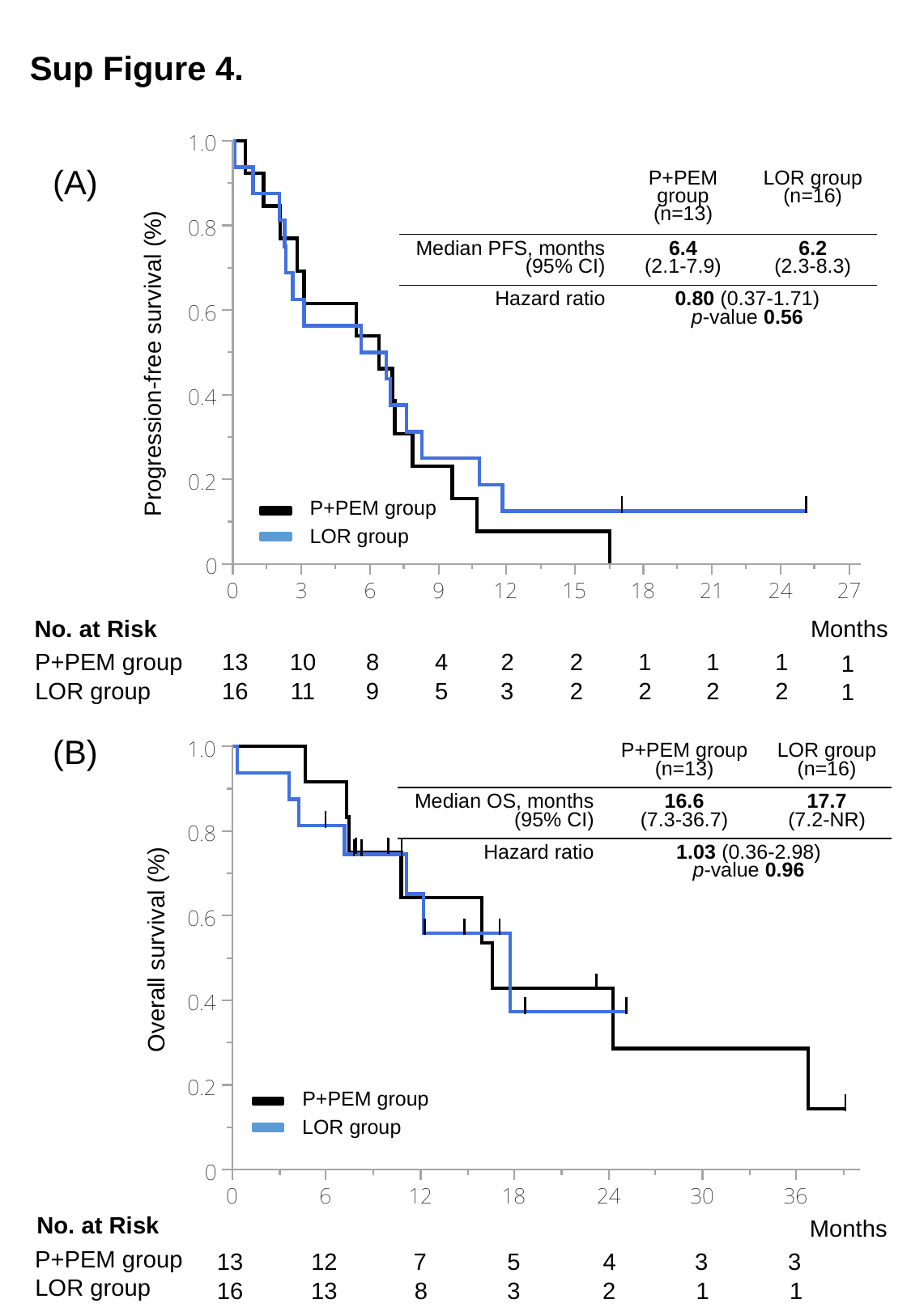

Sup Figure 4.
(A)
| | P+PEM group (n=13) | LOR group (n=16) |
| --- | --- | --- |
| Median PFS, months (95% CI) | 6.4 (2.1-7.9) | 6.2 (2.3-8.3) |
| Hazard ratio | 0.80 (0.37-1.71) p-value 0.56 | |
Progression-free survival (%)
P+PEM group
LOR group
No. at Risk
Months
P+PEM group
13
10
8
4
2
2
1
1
1
1
LOR group
16
11
9
5
3
2
2
2
2
1
(B)
| | P+PEM group (n=13) | LOR group (n=16) |
| --- | --- | --- |
| Median OS, months (95% CI) | 16.6 (7.3-36.7) | 17.7 (7.2-NR) |
| Hazard ratio | 1.03 (0.36-2.98) p-value 0.96 | |
Overall survival (%)
P+PEM group
LOR group
No. at Risk
Months
P+PEM group
13
12
7
5
4
3
3
LOR group
16
13
8
3
2
1
1
