# Supplementary material for: Lorlatinib Versus Pemetrexed-Based Chemotherapy in Patients With ALK-rearranged NSCLC Previously Treated With Alectinib
Source: JTO Clin Res Rep. 2022 Mar 17;3(5):100311. doi: 10.1016/j.jtocrr.2022.100311 (PMC9046446; doi:10.1016/j.jtocrr.2022.100311)
Supplement: Supplementary — Table [file mmc2.docx]

**Sup Table 1. Treatment-related adverse events**

| **Adverse events** | **Lorlatinib (n=16)** | | **Pem-based CTx (N=22)** | |
| --- | --- | --- | --- | --- |
|  | **All grade,**  **n (%)** | **Grade 3 or 4,**  **n (%)** | **All grade,**  **n (%)** | **Grade 3 or 4,**  **n (%)** |
| Hypercholesterolemia  Hypertriglyceridemia  ALT increased  Peripheral neuropathy  Edema  AST increased  Interstitial lung disease  Cognitive disturbance  Dizziness  Anemia  ALP increased  Diarrhea  GGT increased  Anorexia  Nausea  Fatigue  Constipation  Neutropenia  Platelet count decreased  Mucositis oral  Dry skin  White blood cell decreased  Creatinine increased  Dysgeusia  Vomiting  Rash acneiform | 10 (62.5%)  8 (50.0%)  6 (37.5%)  6 (37.5%)  5 (31.3%)  4 (25.0%)  2 (12.5%)  1 (6.3%)  1 (6.3%)  1 (6.3%)  1 (6.3%)  1 (6.3%)  1 (6.3%) | 3 (18.8%)  4 (25.0%)  1 (6.3%) | 7 (31.8%)  1 (4.5%)  1 (4.5%)  7 (31.8%)  5 (22.7%)  1 (4.5%)  17 (77.3%)  15 (68.2%)  8 (36.4%)  8 (36.4%)  4 (18.2%)  3 (18.2%)  2 (9.1%)  2 (9.1%)  2 (9.1%)  2 (9.1%)  2 (9.1%)  1 (4.5%)  1 (4.5%) | 1 (4.5%)  1 (4.5%)  1 (4.5%) |

ALT; alanine aminotransferase, AST; aspartate aminotransferase, ALP; alkaline phosphatase, GGT; gamma-glutamyl transpeptidase, Pem; pemetrexed, CTx; chemotherapy
